# Unveiling the Cutting-Edge Impact of Polarized Macrophage-Derived Extracellular Vesicles and MiRNA Signatures on TGF-β Regulation within Lung Fibroblasts

**DOI:** 10.3390/ijms25137490

**Published:** 2024-07-08

**Authors:** Alvise Casara, Maria Conti, Nicol Bernardinello, Mariaenrica Tinè, Simonetta Baraldo, Graziella Turato, Umberto Semenzato, Alessandro Celi, Paolo Spagnolo, Marina Saetta, Manuel G. Cosio, Tommaso Neri, Davide Biondini, Erica Bazzan

**Affiliations:** 1 Department of Cardiac, Thoracic, Vascular Sciences and Public Health, University of Padova and Padova City Hospital, 35128 Padova, Italy; alvise.casara@phd.unipd.it (A.C.); maria.conti.2@phd.unipd.it (M.C.); nicol.bernardinello@unipd.it (N.B.); mariaenrica.tine@unipd.it (M.T.); simonetta.baraldo@unipd.it (S.B.); graziella.turato@unipd.it (G.T.); umberto.semenzato@aopd.veneto.it (U.S.); paolo.spagnolo@unipd.it (P.S.); marina.saetta@unipd.it (M.S.); manuel.cosio@mcgill.ca (M.G.C.); davide.biondini@unipd.it (D.B.); erica.bazzan@unipd.it (E.B.); 2 Centro Cardiologico Monzino IRCCS, 20138 Milan, Italy; 3 Centro Dipartimentale di Biologia Cellulare Cardiorespiratoria, Dipartimento di Patologia Chirurgica, Medica, Molecolare e dell’Area Critica, Università degli Studi di Pisa, 56124 Pisa, Italy; alessandro.celi@unipi.it; 4 Meakins-Christie Laboratories, Respiratory Division, McGill University, Montreal, QC H3A 0G4, Canada; 5 Department of Medicine, University of Padova, 35128 Padova, Italy

**Keywords:** fibroblasts, TGF-β, macrophages, EVs, miRNAs

## Abstract

Depending on local cues, macrophages can polarize into classically activated (M1) or alternatively activated (M2) phenotypes. This study investigates the impact of polarized macrophage-derived Extracellular Vesicles (EVs) (M1 and M2) and their cargo of miRNA-19a-3p and miRNA-425-5p on TGF-β production in lung fibroblasts. EVs were isolated from supernatants of M0, M1, and M2 macrophages and quantified using nanoscale flow cytometry prior to fibroblast stimulation. The concentration of TGF-β in fibroblast supernatants was measured using ELISA assays. The expression levels of miRNA-19a-3p and miRNA-425-5p were assessed via TaqMan-qPCR. TGF-β production after stimulation with M0-derived EVs and with M1-derived EVs increased significantly compared to untreated fibroblasts. miRNA-425-5p, but not miRNA-19a-3p, was significantly upregulated in M2-derived EVs compared to M0- and M1-derived EVs. This study demonstrates that EVs derived from both M0 and M1 polarized macrophages induce the production of TGF-β in fibroblasts, with potential regulation by miRNA-425-5p.

## 1. Introduction

Fibroblasts, derived from the embryonic mesenchyme, are the main cells that characterize the connective tissue and are extensively involved in many physiological processes, such as organ homeostasis, shaping and maintaining tissue structure through extracellular matrix remodeling. Moreover, fibroblasts play a crucial role in inflammation and wound healing secondary to tissue damage, supporting the recruitment and activation of immune cells and secreting and responding to cytokines, chemokines, and other inflammatory stimuli [1,2,3]. Among the different cytokines produced by fibroblasts, TGF-β holds a key position both enhancing the recruitment of immune-inflammatory cells to the site of inflammation and stimulating extracellular matrix deposition, thus wound healing [4].

Besides their plural physiological functions, when an abnormal fibroblast activation occurs, it can lead to pathological inflammatory and pro-fibrotic processes that are at the basis of various pathological conditions, among which lung diseases are extensively involved [3,5,6,7,8]. Throughout the inflammatory process, tissue injury triggers the activation of macrophages, which in turn release numerous pro-inflammatory mediators. These mediators serve to stimulate the migration, proliferation, and activation of fibroblasts themselves [4]. Consequently, fibroblasts generate a plethora of cytokines that not only regulate the inflammatory response but also contribute significantly to tissue repair [9].

Depending on the local environments, macrophages can be polarized in two subtypes: classically activated macrophages (M1) and alternatively activated macrophages (M2) [10,11,12]. In these pathological processes, fibroblast activation induced by macrophages is crucial, and preliminary findings suggest that extracellular vesicles (EVs) could mediate this cell–cell interaction.

EVs are lipid bilayer-enclosed spheres released by all cell types and are abundantly present in all extracellular fluids (circulating blood, cerebrospinal fluid, and urine). According to the size, origin, and surface markers [13,14], these particles were distinguished into exosomes (the smaller EVs with a diameter < 100 nm), microvesicles (a medium/large EV with a diameter between 100 and 1000 nm), and lastly, apoptotic bodies (with a diameter ≥ 1000 nm) [15,16,17,18].

Recently, the knowledge of EVs and their role in health and disease have increased drastically. These vesicles carry a variety of biological molecules, and they can communicate with local and distant cells, changing the metabolism of these cells [19].

EV cargo can consist of different biological molecules, capable of modulating biological effects, among which microRNAs (miRNA) are key. It is becoming evident that miRNAs (small non-coding RNAs with an average 22 nucleotides in length) play significant roles in regulatory mechanisms operating in various organisms, including developmental timing and host–pathogen interactions as well as cell differentiation, proliferation, apoptosis, and tumorigenesis [20]. miRNAs regulate gene-expression post-transcriptionally by binding to the 3′-untranslated region on messenger RNA (mRNA). Through this, they suppress translation or induce degradation of the target mRNA [21].

Multiple miRNAs could regulate TGF-β production, among which miRNA-19a-3p and miRNA-425-5p play a key role targeting downstream effector SMADs, TGF-β receptors, or TGF-β transcription [22,23,24,25,26,27,28,29].

Hence, in light of the current literature, we can hypothesize that a modulation of miRNA-19a-3p and miRNA-425-5p in macrophage-derived EVs could determine an increase in the levels of TGF-β production on fibroblast target cells, enhancing their pro-inflammatory and pro-fibrotic activity.

The aim of the present study was to evaluate the possible effect of polarized macrophage-derived EVs (M1 and M2) and their miRNA-19a-3p and miRNA-425-5p cargo on the production of TGF-β by lung fibroblasts.

## 2. Results

### 2.1. Production of Macrophage-Derived EVs Quantified by Flow Cytometry

**Figure 1:**
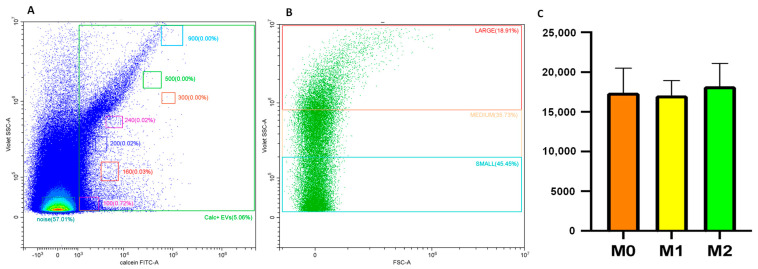
The flow cytometry analysis of macrophages supernatants. (**A**) The violet side scatter (SSC)/Calcein-green fluorescence profile of EVs. Dimensional gates, based on fluorescent bead analysis, were reported as a reference for EV identification. (**B**) The size distribution of Calcein^+^ events based on V-SSC: large (red square), medium (orange square), and small (blue square). (**C**) The production of macrophage-derived EVs quantified by flow cytometry. Orange bar graph = M0-derived EVs; yellow bar graph = M1 derived EVs; green bar graph = M2-derived EVs. The data represent the mean ± standard deviation of the results obtained from three independent experiments.

THP-1 monocytes were differentiated into M0, M1, and M2 macrophages [30]. Macrophage-derived EVs were isolated to stimulate fibroblasts. Before fibroblast stimulation, the EV concentration was quantified by nanoscale flow cytometry. EVs were identified as Calcein^+^ events (Figure 1A,B) and expressed as events/µL.

The total number of EVs (events/µL) was similar in M0, M1, and M2 macrophages. In particular, the concentration of M0 macrophage-derived EVs was 17,398 ± 3331 (events/µL), M1 macrophage-derived EVs was 16,680 ± 1635 (events/µL), and M2 macrophage-derived EVs was 18,223 ± 2766 (events/µL; Figure 1C).

**Figure 2:**
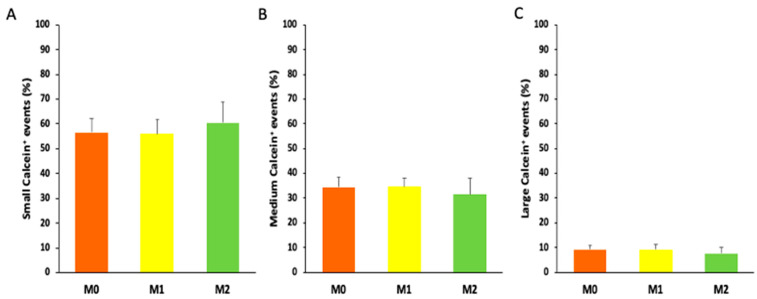
The size distribution of macrophage-derived EVs quantified by flow cytometry. The size distribution of each subset of macrophage-derived EVs ((**A**) small 100–200 nm; (**B**) medium 200–300 nm; (**C**) large 300–900 nm) among the three groups of macrophage-derived EVs (orange bar graph = M0-derived EVs; yellow bar graph = M1-derived EVs; green bar graph = M2-derived EVs). The data represent the mean ± standard deviation of the results obtained from three independent experiments.

An in-depth dimensional analysis allowed us to discriminate three subsets of Calcein^+^ EVs: small (100–200 nm), medium (200–300 nm), and large (300–900 nm). The percentage of small Calcein^+^ EVs was not significantly different between M0 (56.48 ± 5.72%), M1 (55.94 ± 5.73%), and M2 macrophages (60.43 ± 8.56%). The percentage of medium Calcein^+^ EVs was not significantly different between M0 (34.31 ± 4.27%), M1 (34.51 ± 3.41%), and M2 macrophages (31.50 ± 6.35%). Finally, the percentage of large Calcein^+^ EVs was also not significantly different between M0 (9.20 ± 1.49%), M1 (9.20 ± 2.01%), and M2 macrophages (7.57 ± 2.46%) (Figure 2).

### 2.2. TGF-β Production by Human Lung Fibroblasts after Stimulation with Macrophage-Derived EVs

**Figure 3:**
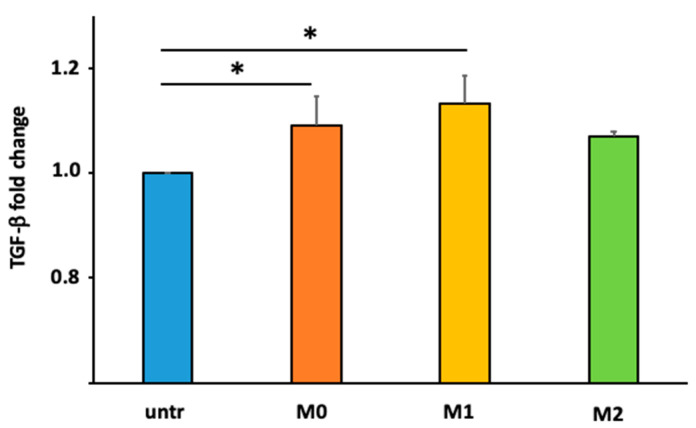
Fold change in fibroblast TGF-β production after medium–large macrophage-derived EV stimulation, normalized on untreated fibroblasts. Light blue bar graph = untreated fibroblasts; orange bar graph = fibroblasts stimulated with M0-derived EVs; yellow bar graph = fibroblasts stimulated with M1-derived EVs; green bar graph = fibroblasts stimulated with M2-derived EVs. The data represent the mean ± standard deviation of the results obtained from three independent experiments. * *p* value < 0.05.

TGF-β levels were evaluated in cell culture supernatants after fibroblast stimulation with medium and large macrophage-derived EVs. The ELISA assays (Figure 3) shows that stimulation with EVs derived from macrophages increased the release of TGF-β by fibroblasts.

In particular, fibroblast stimulation with M0- and M1-derived EVs increased significantly the levels of TGF-β compared to untreated fibroblasts (*p* < 0.05). Conversely, fibroblast stimulation with M2-derived EVs does not increase TGF-β production (*p* = 0.06).

### 2.3. miRNA-425-5p and miRNA-19a-3p Expression in Macrophage-Derived EVs

**Figure 4:**
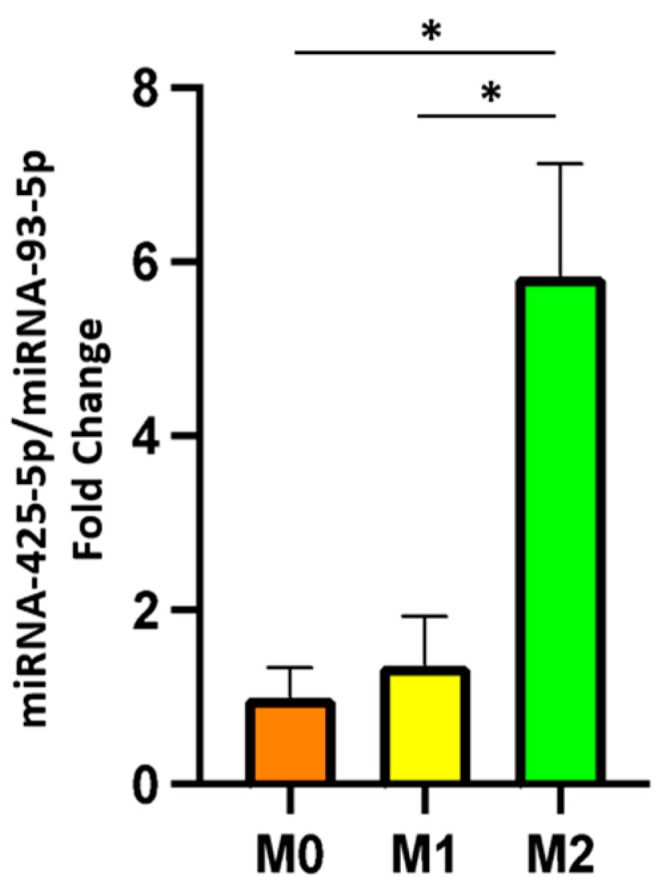
miRNA-425-5p fold change in macrophage-derived EVs. Orange bar graph = M0-derived EVs; yellow bar graph = M1-derived EVs; green bar graph = M2-derived EVs. The data represent the mean ± standard deviation of results obtained from three independent experiments. * *p* value < 0.05.

The relative miRNA-425-5p and miRNA-19a-3p expression levels were investigated in M0, M1, and M2 medium and large macrophage-derived EVs. miRNA-425-5p expression was significantly upregulated in M2-derived EVs compared to M0-derived EVs and M1-derived EVs. Conversely, miRNA-425-5p expression was not significantly different in M1-derived EVs compared to M0-derived EVs (Figure 4).

Finally, miRNA-19a-3p expression was not significantly different in M0-, M1-, and M2-derived EVs.

## 3. Discussion

In recent years, numerous studies have elucidated the intricate cellular and molecular mechanisms underlying fibroblast activation. Among these investigations, significant evidence suggests that this activation is intricately linked to intercellular communication, notably involving macrophages. Particularly within the context of transforming growth factor-beta (TGF-β) production, these studies highlight the pivotal role played by macrophages in orchestrating fibroblast activation. It has become clear that activated macrophages communicate with target cells (i.e., fibroblasts) to exert their immunomodulatory effects through extracellular vesicles (EVs).

Extensively considered as little more than cell waste, several studies have placed EVs at the cutting edge of research into intercellular communication [31]. EVs are a group of membrane-enclosed vesicles that are naturally released by all cell types. Recently, EVs have been recognised as vital information carriers that transfer their cargos from parent cells to recipient cells, modulating the physiological or pathological process. The functions of macrophage-derived EVs have been extensively investigated, while little is known on the effect of polarized macrophage-derived EVs on recipient cells, in particular fibroblasts. EVs’ derived macrophage contents may vary with different macrophage phenotypes or local environments. Depending on the local environments, macrophages can be divided into two subtypes: classically activated macrophages (M1) and alternatively activated macrophages (M2) [10,11,12]. It is known that both subtypes of macrophages are capable of producing EVs, but studies in the literature focus on the effects of a single subtype of EVs [32,33,34,35] rather than comparing the effects of one subtype to the other.

Therefore, in the present study, we investigated whether medium and large EVs derived from polarized macrophages (M1 and M2) are able to stimulate human lung fibroblasts to produce TGF-β. First, using standard stimuli (PMA, IFNγ + LPS, and IL-4), we polarized THP-1-monocytes into three different macrophage subtypes: undifferentiated macrophage M0, classically activated macrophage M1, and alternatively activated macrophage M2, as reported by Baxter and colleagues [30]. After polarization, for the first time, we quantified, by cytofluorimetric analysis, the total number of EVs produced by different macrophage subtypes, and we observed that M0, M1, and M2 produced the same amount of EVs.

Upon noting that the overall number of EVs remained consistent across the three subgroups of macrophages, our curiosity shifted towards exploring the possibility of distinct size distributions among these EVs. It is well known that microvesicles (MVs), which are part of EVs, span a variable size range (100–1000 nm) generated by the shedding of the plasma membrane. In particular, MVs represent a heterogeneous class of vesicles comprising distinct subpopulations that vary in terms of composition and molecular cargo [36,37]. Using cytofluorimetric violet SSC (VSSC) technology, we are able to distinguish three subgroups of microparticles: small, medium, and large. We characterized the size distribution of MVs derived from polarized macrophages, and we found a similar prevalence of small MVs, without statistical significance between the groups. Second, we showed that the size distribution of all subsets of MVs was similar, indicating that the different polarized macrophages produced the same amount of EVs with the same size distribution.

Generally, the activation of fibroblasts may be induced by the cross-talk with macrophages as part of various physiological processes, particularly in the context of tissue repair and immune responses. It is known that macrophages promote the proliferation and activation of fibroblasts, which start producing TGF-β [4,9,38]. As described previously, EVs may play a role in intercellular communication by acting as signaling complexes that directly stimulate target cells, such as fibroblasts, and mediate the transfer of genetic information. EVs from M1 macrophages stimulate extracellular matrix production [32], while EVs from M2 macrophages activate the production of TGF-β and profibrotic factors (e.g., α-SMA, Collagen I) [34,35]. Therefore, it is evident that EVs derived from polarized macrophages exhibit different messages and different types of miRNA.

These results revealed that compared with baseline conditions (untreated), the production of TGF-β in lung fibroblast was higher after stimulation with EVs derived from polarized macrophages. However, this increase was statistically significant only with EVs derived from M0 and M1 but not with M2. To find out why the TGF-β production observed was different, we tried to consider the different content in miRNA biological cargo. We decided to analyze the effect of two specific miRNAs involved in the modulation of TGF-β production: miRNA-19a-3p and miRNA-425-5p [22,23,24,25,26,27,28,29]. These miRNAs are considered negative regulators of TGF-β production, modulating Smad2 signaling or targeting TGF-β Receptor II.

Our results showed higher levels of miRNA-425-5p in M2 compared to both M1- and M0-derived EVs. Conversely, miRNA-425-5p expression levels were not significantly different in M1 compared to M0-derived EVs. This is consistent with the inhibitory function of the miRNA-425-5p and with our observation that both M0 and M1 but not M2 macrophage-derived EVs induced a significant increase in TGF-β production in fibroblasts. These results are also congruent with previous studies that evaluated the role of miRNA-425-5p in different experimental models and that defined miRNA-425-5p as a suppressor of TGF-β signaling pathway [39,40,41].

In contrast with previous reports [22,23,24,25], our analysis of miRNA-19a-3p was not significantly different in the subgroups of macrophage-derived EVs, thus the differences observed in fibroblast TGF-β production are not due to miRNA-19a-3p levels. The complexity of mechanisms that regulate the TGF-β pathway highlight the difficulty in defining the real impact of EV-derived miRNAs, especially considering the numerous molecules and miRNA that EVs can transport. While certain cell types may exhibit limited expression of specific miRNA targets, others might possess an abundance of such targets, leading to the dilution of the miRNAs’ impact.

It is therefore critical to assess the influence of the miRNAs in the context of a specific cell. Furthermore, it is important to underline that the different expression profiles may indicate that the physiological functions of miRNAs could be different in different EV subtypes.

Even though our model could partially explain the possible biological effect of macrophage-derived EVs on lung fibroblast TGF-β production, there are some limitations in the study that ought to be considered. In particular, our study has methodological limitations. EV types were analyzed using high-resolution flow cytometry, and other complementary techniques could be applied to complement this approach and further expand the understanding role of EVs, as recommended by the MISEV guideline. However, we used the next generation of flow cytometers (CytoFLEX Beckman Coulter), which can detect EVs as small as 100 nm, to identify and separate the EV population according to size, as suggested by the MISEV guidelines [15]. We focused our attention on the effect of medium-to-large EVs, but it is possible that smaller EVs, such as exosomes, also affect TGF-β production. Additionally, this study did not evaluate the transformation of fibroblasts to myofibroblasts using specific markers, nor was immunofluorescence used to assess the presence of TGF-β within the cells. The analysis of EV protein expression (i.e., M1 and M2 macrophage population markers) might be investigated to better differentiate M1 and M2 EVs. Due to their role in the TGF-β pathway, we chose to analyze and compare the production of two miRNAs (miRNA-19a-3p and miRNA-425-5p) among the complex and heterogeneous total miRNA cargo of EVs. This analysis could be integrated with an additional miRNA profile study. Finally, to demonstrate a direct link between miRNA levels and TGF-β production, it would be necessary to perform transfection experiments with mimics or antisense miRNAs. Further studies are needed to clarify the role of miRNA-19a-3p and miRNA-425-5p in the lung fibroblast activation and transition.

## 4. Materials and Methods

### 4.1. Cell Cultures: THP-1 and IMR-90

The human monocyte cell line THP-1 was obtained from ECACC (Salisbury, UK) and cultured in standard medium RPMI media (Sigma-Aldrich, St. Louis, MO, USA) 10% Fetal Bovine Serum (FBS), 1% penicillin-streptomycin, and 1% glutamine (Gibco) and maintained at 37 °C, 5% CO_2_ in a humidified tissue culture incubator.

The human lung fibroblast cell line IMR-90 was obtained from ATCC (Manassas, VA, USA) and cultured in standard medium Eagle’s Minimum Essential Medium (EMEM—ATCC) containing 10% Fetal Bovine Serum (FBS, Gibco, ThermoFisher Waltham, MA, USA), 1% penicillin-streptomycin, and 1% glutamine (Gibco). All experiments were performed with cell passage between 4 and 9.

### 4.2. THP-1 Differentiation

THP-1 monocytes were differentiated into resting macrophages (M0), classically activated macrophages (proinflammatory—M1), alternatively activated macrophages (anti-inflammatory—M2) [30]. Cells were counted and seeded in 12 wells at a concentration of 400,000 cells/mL.

THP-1 monocytes are differentiated into resting macrophages (M0) with 24 h incubation with 25 ng/mL phorbol 12-myristate 13-acetate (PMA, Sigma, P8139) followed by 24 h incubation in RPMI medium. After stimulation, cells were washed twice in sterile PBS, and standard medium was replaced for cell resting for 72 h. After 72 h resting, cells were stimulated for 48 h with IFNγ 20 ng/mL (SinoBiological; Eschborn, Germany) + LPS 250 ng/mL (Enzo Life Sciences; New York, USA) for M1 polarization or with IL-4 20 ng/mL for M2 polarization. Surface markers of differentiation were investigated with flow cytometry. Surface markers of differentiation were investigated with flow cytometry. CD80+ was used as a marker for M1 differentiation, and the percentage of CD80-positive macrophages increased 3-fold compared with M0 and M2. CD206 expression was used as M2 marker of differentiation, and the percentage of CD206-positive macrophages increased 3-fold compared with M0 and M1 (Figure S1 Online Data Supplement). After 48 h, supernatant was collected for EV isolation.

### 4.3. EV Characterization and Isolation

Supernatants were collected from differentiated M0, M1, and M2 macrophages and initially centrifuged at 16,000× *g* for 2 min to remove dead cells and big cell fragments. After cell debris removal, the whole supernatant containing small, medium, and large EVs was collected for flow cytometry analysis and EV size characterization. Afterwards, supernatant was then centrifuged at 14,000× *g* for 30 min at 12 °C to pellet and isolate EVs [42,43]. After centrifugation, supernatant was removed, and pelleted medium–large EVs were resuspended in standard complete fibroblast medium to stimulate fibroblasts.

### 4.4. Fibroblast Stimulation

Human lung fibroblasts from cell line IMR-90 were cultured in standard condition. Cells were counted and seeded in 12 wells at a cell density of 20,000 cells/cm^2^. Cells were starved in a serum-free medium for 24 h and then stimulated for 48 h with fibroblast complete medium containing resuspended medium and large EVs derived from M0, M1, or M2 polarized macrophages (about 7000–8000 events/μL). Afterwards, supernatant was collected for ELISA analysis. All experiments were performed with cell passage between 3 and 6.

### 4.5. Elisa

TGF-β secretion in the culture medium was assayed using an ELISA kit according to the procedure recommended by the supplier’s protocols ((RayBio^®^ Human TGF-beta 1 ELISA Kit, RayBiotech; Peachtree Corners, GA, USA). Each experiment was conducted in triplicate.

### 4.6. Flow Cytometry

Flow cytometry analysis of EVs in the supernatant samples were performed using a CytoFLEX flow cytometer (Beckman Coulter; Brea, CA, USA). For EV size calibration of the flow cytometer, fluorescent polystyrene beads, Gigamix in a mix 1:1 of Megamix FSC & SSC Plus (BioCytex, Marseille, France), were used in sizes of 0.1, 0.16, 0.2, 0.24, 0.3, 0.5, and 0.9 μm. Violet side scatter (VSSC) and FL1 channel gain were set to visualize the beads. The side scatter (SSC) from the 405 nm violet laser (VSSC) was used as a trigger signal to discriminate the noise. Gigamix bead solution was gated excluding background noise. After turning the set in VSSC and forward scatter (FSC), a rectangular gate was set between the 0.1 and 0.9 μm bead populations and defined as EV gate. To determine EV concentration, twenty microliters of macrophage supernatants were stained with 10 μL of calcein-AM (Sigma-Aldrich) and incubated 30 min at 37 °C. Buffer controls without EVs, detergent lysis treatment performed by incubating the filtered PBS-diluted EVs samples in 1% Triton™ X-100 for 30 min at RT, and unstained samples were included in all analyses as technical controls [44]. To confirm that EVs detected by flow cytometry were lipid membrane vesicles, the samples were treated with 1% Triton X-100 (Sigma-Aldrich), a lipid solubilizing detergent, for 10 min at room temperature (RT) and compare with the non-treated samples (Figures S2 and S3 Online Data Supplement) [45,46,47]. Files were exported, and data were evaluated with CytExpert (Software Version 1.2, Beckman Coulter).

### 4.7. miRNA Extraction and TaqMan miRNA Assay

Total microRNAs (miRNAs) were isolated from macrophage-derived EVs following the manufacturer’s instructions using Total Exosome RNA and Protein isolation kit (Invitrogen 4478545). Once extracted, cDNA templates were prepared following the manufacturer’s instructions using TaqMan Advanced miRNA Assays (applied biosystems A25576). Briefly, poly (A) tailing reaction of mRNA was followed sequentially by adaptor ligation, reverse transcription, and miR-Amp reactions using manufacturer’s protocols. On the cDNA templates obtained, qPCR was performed to evaluate hsa-miRNA-19a-3p expression (mir 479228 sequence: UGUGCAAAUCUAUGCAAAACUGA), hsa-miRNA-425-5p expression (mir 478094 sequence: AAUGACACGAUCACUCCCGUUGA). miRNA expression was calculated using the ΔΔ-threshold cycle (Ct) method and normalized to miRNA-93-5p (mir 478210 sequence: CAAAGUGCUGUUCGUGCAGGUAG) used as an endogenous normalizer.

### 4.8. Statistical Analysis

The data are presented as the mean  ±  standard deviation. For continuous variables, normal distributions were tested using the Shapiro–Wilk test. One-way ANOVA with Turkey’s multiple comparison test was used to evaluate the parametric data. The Kruskal–Wallis test and Dunn test for multiple comparison were used to evaluate the non-parametric data. All data were analyzed with SPSS statistical software (version 3.5.2). *p* < 0.05 was considered statistically significant.

## 5. Conclusions

In conclusion, our investigation into the impact on TGF-β production by lung fibroblasts stimulated with EVs derived from polarized macrophages highlight the intricate mechanisms of intercellular communication in physiopathological processes. In particular, these results showed that EVs produced by polarized macrophages are able to stimulate lung fibroblasts to produce TGF-β. In our model, we observed a significant upregulation of miRNA-425-5p in M2-derived EVs and an associated increase in TGF-β release by fibroblasts. These findings may suggest a potential involvement of the miR-425-5p/TGF-β axis in fibroblast activation, which warrants further investigation through functional studies to confirm this regulatory mechanism. Additionally, studies on EV-mediated lung fibroblast activation are needed to better characterize the contribution of EVs to fibrotic mechanisms and to elucidate their potential as therapeutic targets and biomarkers.

## Data Availability

The data presented in this study are available on request from the corresponding author.

## Supplementary Materials

The supporting information can be downloaded at https://www.mdpi.com/article/10.3390/ijms25137490/s1.
