# Supplementary material for: Unveiling the Cutting-Edge Impact of Polarized Macrophage-Derived Extracellular Vesicles and MiRNA Signatures on TGF-β Regulation within Lung Fibroblasts
Source: Int J Mol Sci. 2024 Jul 8;25(13):7490. doi: 10.3390/ijms25137490 (PMC11242851; doi:10.3390/ijms25137490)
Supplement: Supplementary file 1 [file ijms-25-07490-s001.zip › ijms-3017484-supplementary.pdf]

Unveiling the Cutting-Edge Impact of Polarized Macrophage-Derived Extracellular Vesicles and MiRNA Signatures on TGF-β Regulation within Lung Fibroblasts

Alvise Casara[1†], Maria Conti [1,2†], Nicol Bernardinello[1], Mariaenrica Tinè[1], Simonetta Baraldo[1], Graziella Turato[1], Umberto Semenzato[1], Alessandro Celi[3], Paolo Spagnolo[1], Marina Saetta[1], Manuel G. Cosio[1,4], Tommaso Neri[3], Davide Biondini[1,5‡] and Erica Bazzan[1‡].*

Online Data Supplement

Figure S1

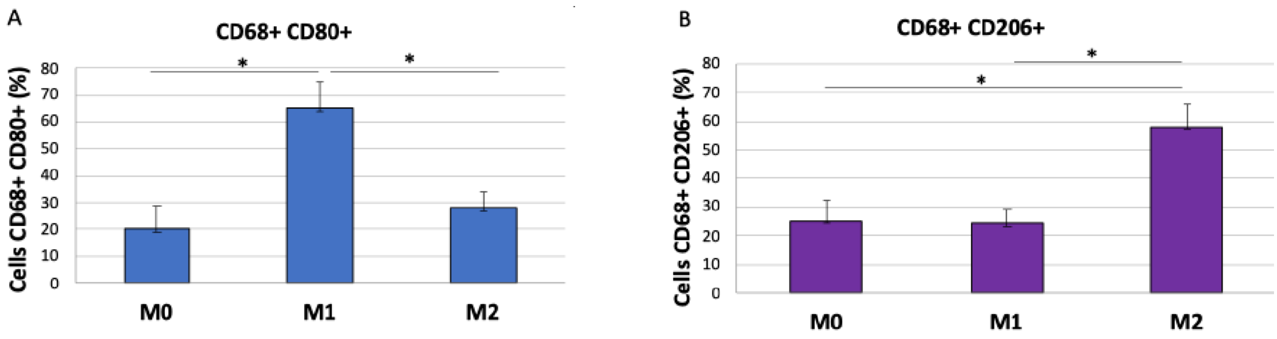

Figure S1. Percentage of polarized macrophages. Expression of CD80 (panel A) and CD206 (Panel B) from M0, M1 and M2 macrophages. Bars represent the mean (SEM) of 3 independent experiments. * p<0.005 for all comparison.

Figure S2

A

B

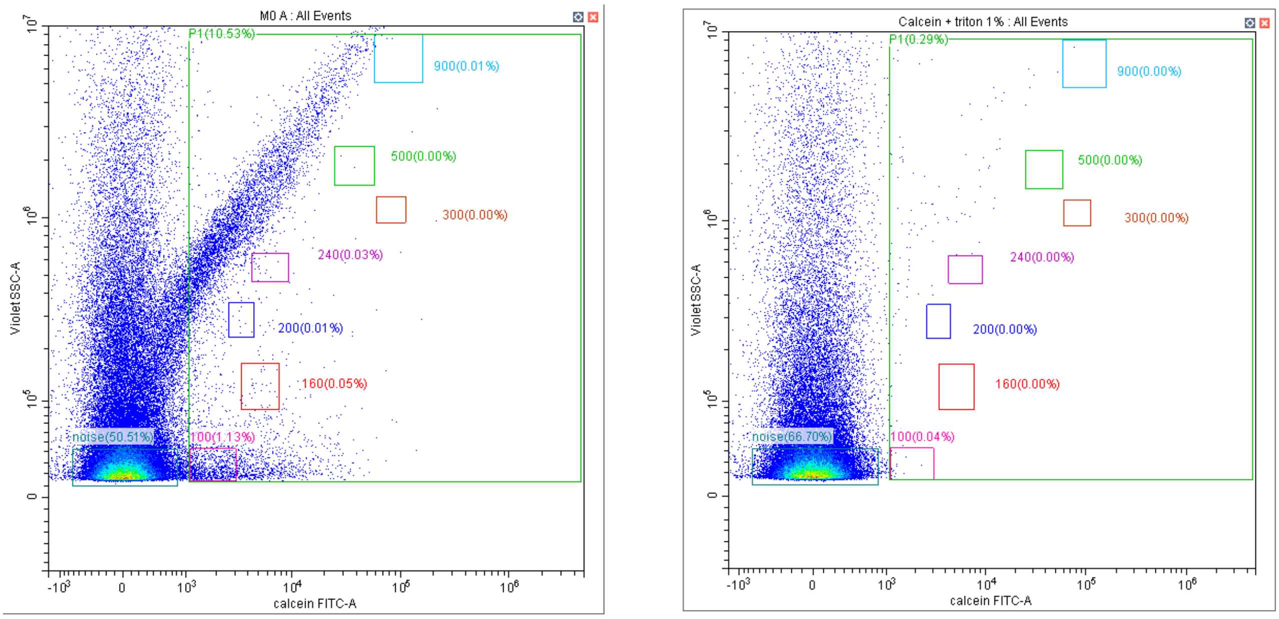

Figure S2: To confirm that EVs detected by flow cytometry were lipid membrane vesicles the samples were treated with 1% Triton X-100 for 10 min at RT (B) and compared with the non-treated

samples (A). Permeabilized/lysed EVs do not label with calcein-AM that allows differentiation between intact EVs and debris.

Figure S3

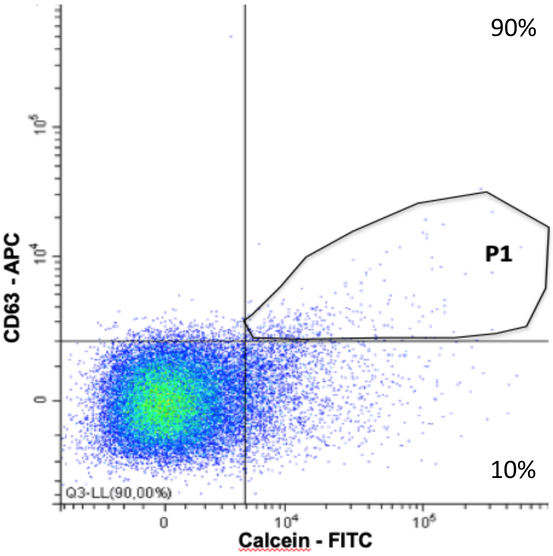

Figure S3. Flow cytometry plot demonstrating Calcein AM+ CD63+ EVs events (gate P1). Data shows that only 10% of our total EVs-Calcein AM+ were also CD63+.
